# Colorectal Cancer Screening: The Role of Psychological, Social and Background Factors in Decision-making Process

**DOI:** 10.2174/1745017901814010063

**Published:** 2018-03-21

**Authors:** Giulia Cossu, Luca Saba, Luigi Minerba, Mario Mascalchi

**Affiliations:** 1 Department of Medical Sciences and Public Health, University of Cagliari, Cagliari, Italy; 2 Department of Radiology, AOU, University of Cagliari, Cagliari, Italy; 3 Department of Experimental and Clinical Biomedical Sciences, University of Florence, Florence, Italy

**Keywords:** Cancer screening, Compliance, Barriers, Cancer fear, Cancer worry, Health anxiety, Embarrassment, Risk perception, Colorectal cancer

## Abstract

Since ColoRectal Cancer (CRC) remains the third cause of cancer death in the world, a better understanding of the reasons underlying poor adherence to and delay in undergoing CRC screening programs is important.

CRC screening decision-making process can be conceptualized as the relationship between intention and behavior and needs to be investigated including the impact on patients’ decision of a broad range of psychological factors and personal predisposition as fear of a positive screening test, poor understanding of the procedure, psychological distress, anxiety, anticipation of pain, feelings of embarrassment and vulnerability. Also socioeconomic, ethnic and sociological influences, and organizational barriers have been identified as factors influencing CRC screening adherence. Decision-making process can finally be influenced by the healthcare background in which the intervention is promoted and screening programs are carried out.

However, there is still a gap on the scientific knowledge about the influences of diverse elements on screening adherence and this deserves further investigations in order to carry out more focused and effective prevention programs.

## 1 COLORECTAL CANCER SCREENING TESTS

Although its incidence and mortality have decreased over the past 20 years, ColoRectal Cancer (CRC) remains one of the leading causes of mortality and morbidity in the world [1, 2]. According to guidelines, CRC screening tests are strongly recommended starting from 50 years of age for average risk individuals [3-5] and should continue up to age 75 [6].

The options currently offered for CRC screening include Fecal Occult Blood Test (FOBT), Flexible Sigmoidoscopy (FS), Optical Colonoscopy (OC) and the recently proposed CT Colonography (CTC). FOBT is probably the least expensive screening test and is also well accepted with participation rates of 48% to 63%. However, it has some limitations, such as the need to repeat the test every one/two years and the low sensitivity for large adenomas (≥10 mm) [7]. In spite of being the most complete diagnostic test for CRC and colorectal adenomas, OC shows low acceptance as a screening test. CTC is a highly sensitive and minimally invasive procedure for the detection of cancer (96%) and large adenomas (90%) and has a low risk of complications [7]. The relationship between cost and effectiveness of CTC has been recently debated in order to assess whether and how to include it in the screening programmes [8-13]. CTC is expensive and requires the patient’s ability to complete a successful preparation similar to colonoscopy. However, its main drawback is the radiation dose delivered to the patient [14]. In particular, exposure to ionizing radiations has emerged as a general obstacle to participation in radiological screening of cancers, that is associated with practical and emotional barriers related to the fear of radiation [15, 16]. We carried out a computerized search on Google and PubMed database combining the search terms “cancer screening”, “compliance barriers” and “colorectal cancer” to assess studies that have tried to identify the main adherence barriers to CRC screening programs.

## 2 REASONS FOR NON-ADHERENCE TO COLORECTAL CANCER SCREENING

Despite evidence suggesting that 90% of CRC incidence and 60% of deaths can be prevented through screenings [17, 18], 38% of adults aged 50 years and older have never had a sigmoidoscopy/colonoscopy and 79% have never had a FOBT [19]. Better understanding of the reasons for poor adherence and delay in undergoing screening programs is important in the context of the significant challenge represented by the reduction of CRC incidence and morality [3, 18]. Usually, the CRC screening strategies include FOBT as first exam and OC as further work-up procedure. As it has been underlined, colonoscopy participation rates can be significantly lower than FOBT, and even when FOBT is positive, some patients can refuse to continue the screening process declining OC [20, 21].

Reasons underlying non-adherence to CRC screening are diverse and include socioeconomic, ethnic and sociological influences [22], organizational barriers [23, 24] and General Practitioner (GP) endorsement bent [25]. Also a male gender, long waiting time for appointment and psychiatric illnesses have been reported as factors that are implicated in colonoscopy non-adherence [26-28].

However, a lower educational level has been found to be associated with low colonoscopy compliance. Finally, obese patients have been shown to be more likely to be non-compliant than non-obese patients [29]. Therefore, it is necessary that promotional CRC screening activities take into greater account these factors that predispose some types of patients to poor adherence.

However, there seems to be a gap in the scientific literature especially on the psychosocial factors that might influence patient’s decision to undergo or refuse CRC screening. In particular, knowledge of the psychological effects on participating in CRC screening is limited and there are few studies, with mixed and contradictory results, that have tried to aassess which psychological aspects such as fear can affect the probability that patients undergo specific screening and how much participants may experience psychological stress from participation even after a long time [30].

## 3 PSYCHOLOGICAL FACTORS

In general, fear of a positive test, poor understanding of the procedure, psychological distress, anxiety, anticipation of pain, feelings of embarrassment and vulnerability can determine a poor adherence to cancer screening [23, 31, 32].

However, according to some studies, cancer fear itself can act both as a facilitator and a deterrent to screening participation for breast [33, 34], prostate [34] and ovarian cancer [35] cancers. According to some Authors, cancer fear could promote adherence to screening since the latter can be viewed as a means for seeking reassurance, while for other Authors, cancer fear would be associated with elusive or fatalistic views that are deterrent to screening [36-38]

Regarding CRC screening, fear before undergoing screening was associated with higher adherence, while fear during screening was associated with lower adherence in a sample of elderly adults in Singapore [39] and in a sample of Hispanic Americans [40]. However, this ambivalent relationship between cancer fear and adherence to CRC and other cancer screening was not found in other studies [41, 42]. The main factor that would require greater insight seems to be how fear can lead to a specific behavioral response facilitating, hindering or delaying people from undergoing the screening test.

One possible explanation for the above inconsistencies could be the low uniformity and accuracy of tools used to measure and examine “*cancer fear”* in its different aspects. Usually, history of psychiatric illness, defined as mood, anxiety, and/or psychotic [28] or panic [43-45] disorder, has shown a potential correlation with low adherence to screening and has been assessed using standard instruments.

However fear of undergoing medical examinations and of being affected by specific pathologies may include other psychological features as worry of receiving a positive screening test result, discomfort associated with the invasiveness of the screening tests, some cognitive and affective factors and anxiety predisposition. Accordingly, it is reasonable to assume that specific tools need to be developed considering all the diverse psychological elements that can be involved. In general, the fear of receiving a CRC diagnosis needs to be better understood as a multi-dimensional construct with its cognitive, biological, affective and behavioural components [46, 47]. A review that took into account inhomogeneous definitions and measurement strategies has pointed out that ‘worry about cancer’, ‘cancer-related distress’, ‘intrusive and avoidant thoughts about cancer’, and ‘effects of cancer-related thoughts on mood and daily activities’ are some of the main elements with which the fear of cancer has been operationalised [38]. Three aspects of cancer fear: cognitive, affective and psychobiological aspects and their association with different behavioural effects [47]. It has also been observed that cognitive/affective aspects of cancer fear (cancer as greatest health fear, worry about cancer) may exert their effects at different stages in the decisional process. They were significant facilitators of screening intention because of the desire of being reassured, while to think about cancer can lead to a more visceral negative response that can be a deterrent at the action stage. As outlined by other Authors, the relationship between intention and behaviour needs to be better understood as well as the role of factors that can influence intention and action [48].

## 4 HEALTHCARE BACKGROUND FACTORS

Decision-making process can also be influenced by the healthcare background in which the intervention is promoted and how preventive measurements are presented and screening actions are performed. Full knowledge of the different CRC screening examinations available and their different invasiveness level can also be a relevant factor for adherence. However, research results are not yet exhaustive on these aspects and further studies are required.

Since the CRC screenings remain an elective and voluntary medical procedure and the rate of deaths attributable to low rate of compliance to CRC screening are not negligible, some studies have been carried out with the aim to evaluate efficacy of some preventive health measures. For instance, patient navigation and community health liaisons were developed and trained to guide candidates through screening colonoscopy using a direct endoscopy referral system [49]. They proved to be effective in helping patients to overcome barriers to CRC screening, but with an effect of small size [50, 51].

For this reason, both decision aids and patient navigation have been tested as complementary interventions for promoting CRC screening adherence by influencing the decision process both on a practical/behavioral and cognitive level including patients awareness, knowledge and preferences [52]. Increasing the patient’s knowledge using advanced visual media through local campaigns was found to be associated with higher acceptance rates for screening colonoscopies [53]. Moreover, a community-focused education program has been evaluated as a potential prevention action to reduce poor adherence and make the patient an active participant in their care [54]. The program has provided instruction on CRC prevention and screening through flyer distribution, newspaper advertisements, radio advertisements, and publicly displayed poster to allow patients to keep information and make an appointment showing increasing compliance [54]. Culturally, targeted educational programs about CRC for Blacks and Latinos including education about screening, peer testimony given by a colonoscopy-adherent person, and pre- and post-knowledge assessment have also been realized [55].

Colonoscopy is one of the main CRC screening tests that requires compliance to the procedure, as well as the ability to complete a successful colonoscopy preparation. For this reason, some authors have assessed the role that communication and understanding of basic health information during the process can have for patients adherence. The use of an educational pamphlet has been found to reduce the anxiety levels before colonoscopy, to determine a better colon preparation and to have effect on the quality and the amount of medications used during the procedure [56]. A web-based multimedia program illustrates to patients information material before colonoscopy can increase knowledge, decrease anxiety, procedure time and medication requirements [57].

Since colonoscopy is usually done with sedation to reduce anxiety and discomfort in patients during the examination, some studies have evaluated the capability of listening to music, as an alternative method to reduce pain during the procedure [58-60]. Other alternative methods, such as showing a relaxing video, have been tested in order to evaluate their effects on patient anxiety, pain and experience during colonoscopy with an improved satisfaction especially among patients with a high pre-procedural anxiety score [61]. Finally, in order to evaluate possible benefits of hypnosis during colonoscopy, some authors performed a pilot study selecting the time for procedure, number of vasovagal events, and recovery time as outcomes [62]. The results have suggested that hypnosis can be an effective method to manage anxiety and pain associated with colonoscopy and can reduce the need for sedation, recovery time and vasovagal events.

## CONCLUSION

The CRC screening decision-making process conceptualized as the relationship between intention and behaviour needs to be better understood by examining the impact of a broad range of factors on screening adherence and future prevention programs. In particular, since there is a gap on the scientific knowledge about psychological factors and personal predisposition elements underlying patients’ decision, further studies of these elements are necessary.

It is also necessary that studies take also into consideration the environmental factors that can interfere or facilitate the decision-making process and their specific peculiarities including healthcare background and the characteristics of families, communities, cities and neighborhoods where the interventions are promoted and screening programs are carried out.
